# Finding Sigma-Controlled Promoters

**DOI:** 10.1371/journal.pbio.0040013

**Published:** 2005-12-20

For bacteria, a change in the environment often causes physiological stress. Bacteria cope with that stress by altering the expression of suites of genes, and manufacturing new proteins, which may allow the cell to repair damage or protect itself in the future. This stress-induced gene expression response is often mediated by proteins called sigma factors. Sigma factors bind to the gene-transcribing machinery and direct this machinery to the promoter sites of the target genes. Identifying the binding sites for sigma factors that direct stress responses thus provides an important window on understanding how bacteria behave. Unfortunately, sigma factor binding sequences (otherwise known as promoters) vary from gene to gene, and current identification methods are tedious and prone to error. In a new study, Virgil Rhodius, Carol Gross, and their colleagues describe a novel model that finds these sites quickly and accurately. Their results show that in Escherichia coli bacteria, the main targets for one type of sigma factor, called sigma factor E, are genes that increase production of cell envelope components (inner membrane, periplasm, and outer membrane).

The authors first found sigma factor E sites the hard way—by comparing gene expression in two strains of E. coli that differed in their intrinsic level of sigma E–induced activity. They looked for genes whose expression in the high sigma E activity strain differed most from genes in the low sigma E activity strain, and then searched upstream of these genes for promoters containing these sigma sites (the region to which the transcription machinery binds). This method, called expression profiling, led them to 28 genes. This formed a “starter set,” which could be used to make their model.

By determining the nucleotide sequences and spatial arrangements that were most common at these sites, Rhodius et al. constructed a “position weight matrix,” a prediction tool with which to discover and analyze putative sigma E sites on other genes. Applied to the entire genome of bacterium E. coli K-12, the matrix identified 553 potential sites, which included 27 of the 28 sites identified through expression profiling. However, most of these sites were likely to be false positives. A series of increasingly stringent selection rules was then applied to eliminate those sites that were likely to arise by chance alone, whittling the list down to 39, including 24 of the original 28 sites. Of these 39, the authors confirmed that 37 were actual sigma factor E sites. Using a variety of other screening methods, they determined that the K-12 genome actually contained a total of 49 sigma E binding sites. [Fig pbio-0040013-g001]

**Figure pbio-0040013-g001:**
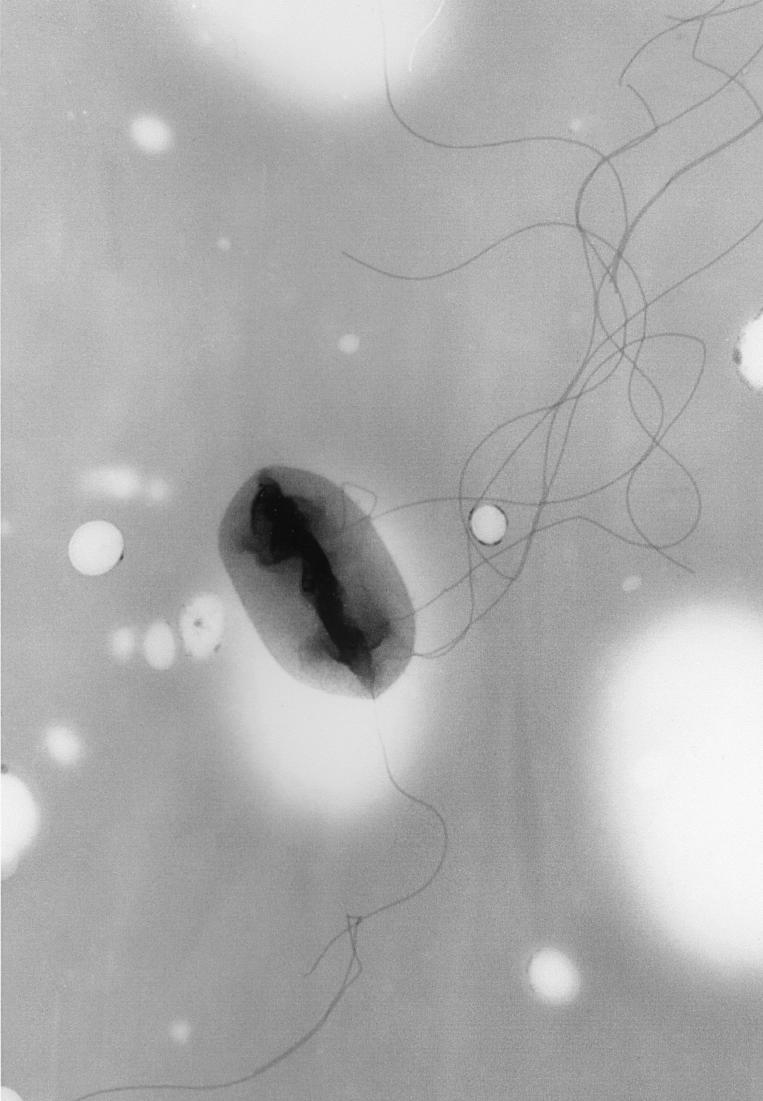
Escherichia coli (visualized with transmission electron microscopy, above) was used as a model system to predict the regulatory DNA targets of sigma factors, bacterial proteins induced by stress. Image: CDC/Elizabeth H. White, M.S

So how good are these results? A predictive model such as this is judged by two measures: sensitivity and precision. Sensitivity, the ratio of validated predictions (“hits”) to total actual sites, indicates how well the model finds true positives. Precision, the ratio of validated predictions to total predictions (hits plus misses), indicates how well the model screens out false positives. A model that claimed that every sequence was a promoter would indeed identify all the real ones, and have a sensitivity of 100%, but the rate of false positives would make the model useless. Similarly, a model so conservative that it only made predictions guaranteed to be right would have a precision of 100%, but might predict so little as to be of no use either.

The Rhodius et al. model has a sensitivity of 37/49, or 76%, and a precision of 37/39, or 95%, in E. coli K-12. The average of these two represents the total performance, or accuracy, of the predictive model, and was 85%, considerably better than previous models.

Finally, the authors used their results to predict the genes activated by sigma E in response to stress in a variety of bacteria. They found that the genes most commonly activated by sigma E primarily affect the integrity of the outer membrane, through promoting the synthesis, assembly, and homeostasis of the two major components of the membrane: lipopolysaccharide and proteins called porins. Other regulated genes promote pathogenic behavior of the bacteria. Experimental validation of the predictions in two different species of bacteria indicated that the model performed well, having a precision of about 75%. Improved ability to find such genes and to understand how they are activated has clear potential for reducing the burden of bacterial infections.—*Richard Robinson*

